# Lysine Acetylation/Deacetylation Modification of Immune-Related Molecules in Cancer Immunotherapy

**DOI:** 10.3389/fimmu.2022.865975

**Published:** 2022-05-02

**Authors:** Peng Ding, Zhiqiang Ma, Dong Liu, Minghong Pan, Huizi Li, Yingtong Feng, Yimeng Zhang, Changjian Shao, Menglong Jiang, Di Lu, Jing Han, Jinliang Wang, Xiaolong Yan

**Affiliations:** ^1^ Department of Thoracic Surgery, Tangdu Hospital, The Air Force Military Medical University, Xi’an, China; ^2^ Department of Medical Oncology, Senior Department of Oncology, Chinese People'’s Liberation Army of China (PLA) General Hospital, The Fifth Medical Center, Beijing, China; ^3^ State Key Laboratory of Cardiovascular Disease, Fuwai Hospital, National Center for Cardiovascular Diseases, Chinese Academy of Medical Sciences, Peking Union Medical College, Beijing, China; ^4^ Department of Outpatient, PLA Rocket Force Characteristic Medical Center, Beijing, China; ^5^ Department of Ophthalmology, Tangdu Hospital, The Air Force Military Medical University, Xi’an, China; ^6^ Department of Thoracic Surgery, 1st Affiliated Hospital of Anhui Medical University, Hefei, China

**Keywords:** acetylation, deacetylation, HAT, HDAC, Cancer, immunotherapy

## Abstract

As major post-translational modifications (PTMs), acetylation and deacetylation are significant factors in signal transmission and cellular metabolism, and are modulated by a dynamic process *via* two pivotal categories of enzymes, histone acetyltransferases (HATs) and histone deacetylases (HDACs). In previous studies, dysregulation of lysine acetylation and deacetylation has been reported to be associated with the genesis and development of malignancy. Scientists have recently explored acetylation/deacetylation patterns and prospective cancer therapy techniques, and the FDA has approved four HDAC inhibitors (HDACi) to be used in clinical treatment. In the present review, the most recent developments in the area of lysine acetylation/deacetylation alteration in cancer immunotherapy were investigated. Firstly, a brief explanation of the acetylation/deacetylation process and relevant indispensable enzymes that participate therein is provided. Subsequently, a multitude of specific immune-related molecules involved in the lysine acetylation/deacetylation process are listed in the context of cancer, in addition to several therapeutic strategies associated with lysine acetylation/deacetylation modification in cancer immunotherapy. Finally, a number of prospective research fields related to cancer immunotherapy concepts are offered with detailed analysis. Overall, the present review may provide a reference for researchers in the relevant field of study, with the aim of being instructive and meaningful to further research as well as the selection of potential targets and effective measures for future cancer immunotherapy strategies.

## Introduction

Post-translational modifications (PTMs) are associated with a large number of cellular processes and regarded as indispensable in maintaining protein function *via* regulating the activity, stability and localization of proteins (1). As major PTMs, deacetylation and acetylation processes are significant factors in signaling and cellular metabolism (2). The real-life processes of histone acetylation and deacetylation are often regulated by histone deacetylases (HDACs) and histone acetyltransferases (HATs), which together participate in numerous dynamic processes and in a wide range of substrate properties such as protein-DNA interactions, transcriptional and metabolic activities, and cell cycle regulation (3). Notably, since a number of significant processes in biology require histone acetylation and deacetylation, such as diabetes, infectious diseases and cancer, the dysregulation thereof is involved in various diseases (4).

In recent decades, owing to the non-negligible effects of acetylation and deacetylation on tumor cell growth and progress, an increasing amount of scientists have investigated acetylation/deacetylation patterns and potential cancer treatment methods (5, 6). Emerging evidence has indicated that suppression of HDACs can potentially inhibit growth and facilitate the differentiation of tumor cells, thereby producing an anticancer effect in HDACs (7). Four compounds (Romidepsin, Belinostat, Panobinostat and Vorinostat) have already been ratified by the Food and Drug Administration (FDA) as HDAC inhibitors (HDACi) for clinical use, with numerous others currently undergoing clinical assessment (8, 9). In the clinical treatment of tumors, immune checkpoints (for instance, CTLA-4, TIM-3, LAG-3, and most importantly, PD-1/PD-L1) have been demonstrated to be effective targets in cancer immunotherapy due to the ability thereof to regulate immune responses (10). Using HDACi alone or in combination with other therapeutic agents such as immune checkpoint inhibitors (ICIs) has been revealed to be effective in cancer immunotherapy, and the significance of acetylation/deacetylation modulation in cancer has attracted much research attention (11).

In the present study, relevant achievements in the field of lysine acetylation/deacetylation modification in cancer immunotherapy were analyzed. First, a brief explanation of the acetylation/deacetylation process and relevant indispensable enzymes that participate therein is provided. Subsequently, a multitude of specific immune-related molecules involved in the lysine acetylation/deacetylation process in the context of cancer are listed, in addition to several therapeutic strategies associated with lysine acetylation/deacetylation modification in cancer immunotherapy. Finally, numerous prospective research fields related with cancer immunotherapy concepts are offered with detailed analysis. The present study may serve as a reference for researchers in the relevant field of study, with the aim of being instructive and meaningful to further research as well as the selection of potential targets and effective measures for future cancer immunotherapy strategies.

## Lysine Acetylation/Deacetylation Process

**Figure 1:**
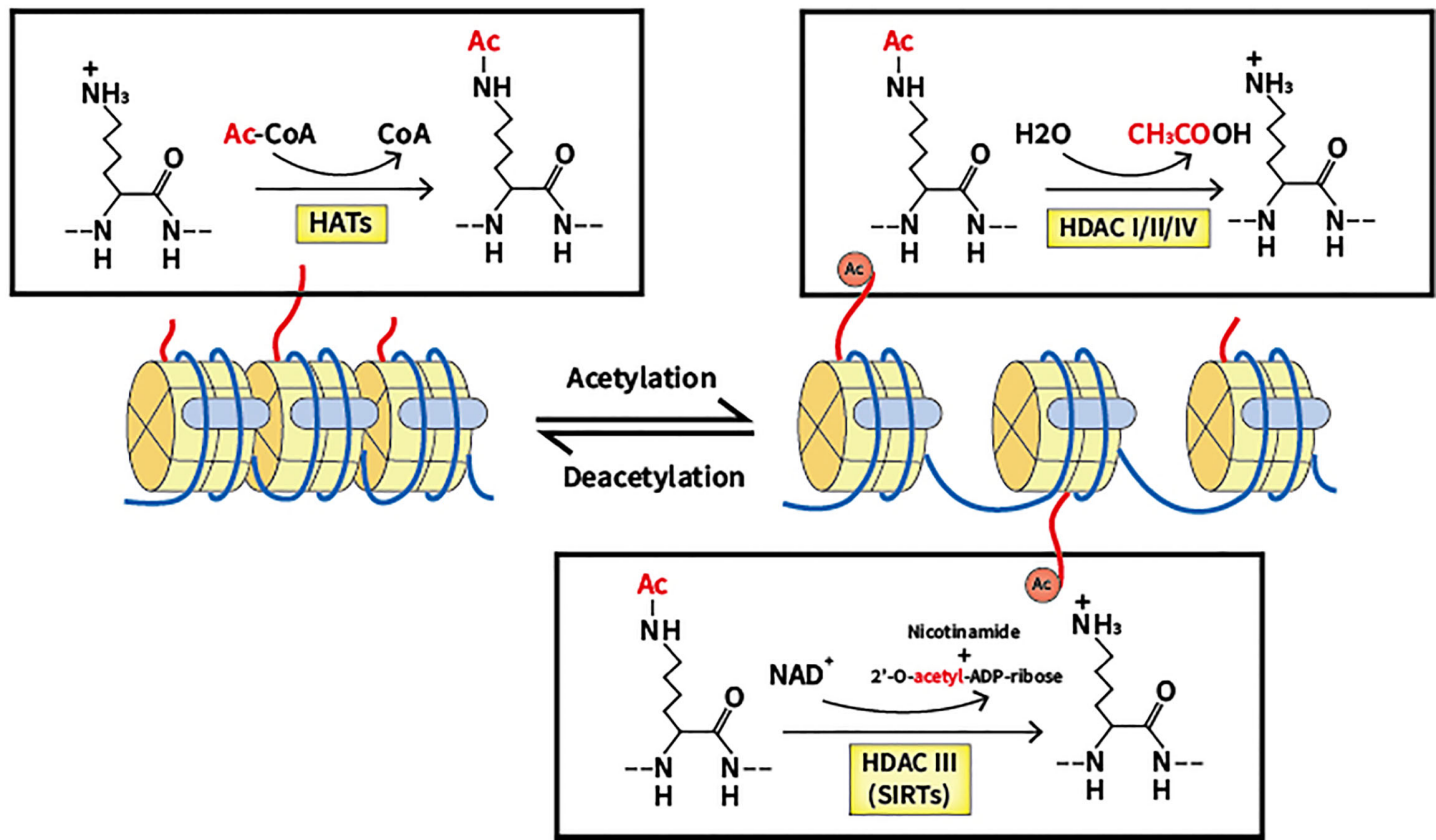
Lysine acetylation/deacetylation process that mediated by HATs and HDACs. HATs, histone acetyltransferases; HDACs, histone deacetylases; SIRTs, sirtuins.

Acetylation was discovered in 1963 and has since been extensively investigated by scientists, being regarded as a crucial PTM in cells (12). By neutralizing the positive charge of acetylated lysine sites of histones, which regulates the mutual effect between targeted histones and the DNA skeleton with negative charge, acetylation modulates and relaxes the chromatin structure (13). In recent decades, a number of substrates have been reported as substrates of acetylation in addition to histone, reflecting the universality of acetylation in cellular processes (14). At the same time, a myriad of proteins with bromodomain like transcription factors can be used to identify lysine acetylation marks, which activates the next step of the transcriptional process (15). Deacetylation is the reverse process of acetylation, and is able to create a tighter connection between histone and DNA, causing a more condensed structure of chromatin that blocks the subsequent transcriptional process (16). Notably, lysine deacetylation appears to be a significant factor in chromatin reconstruction, further influencing gene expression and cellular processes (refer to **Figure 1**).

Two groups of essential enzymes are associated with lysine deacetylation and acetylation: histone deacetyltransferases (HDACs) and histone acetyltransferases (HATs). HATs are enzymes that help move the acetyl group from the acetyl coenzyme A to the lysine N-terminal, which removes the basic charge from the unmodified lysine residue, thereby accelerating the resulting active transcription process (17). According to location, HATs can be classified into two types. In the nucleus, Type A HATs are a group of enzymes that facilitate the transcription process by acting as transcriptional activators, which is the common functional feature thereof. The described enzymes can be divided into three separate families depending on the different substrates and functions: the Gcn5-related N-acetyltransferase (GNAT) family, the p300/CREB-binding protein (CBP) family, and monocytic leukemia zinc finger protein, Ybf2/Sas3, Sas2 and Tip60 (MYST) family (18). The p300/CBP family consists of p300 and CBP, which mainly target H3 and H4 residues. The GNAT family consists of PCAF, Gcn5, Hat1, Hpa2 and Elp3, which mainly act on histones. The MYST family includes Tip60, MOF, Esa1, Sas2, Sas3, Hbo1 and MORF, which mainly target H4 residues (19). Acetylation of newly translated histones is associated with cytoplasmic Type B HATs, and such process is essential for the passage of newly synthesized histones across the nuclear membrane into newly replicated DNA. HatB3.1, Rtt109, HAT1, HAT2, and HAT4 are all types of B-type HATs (20). As well as histones, non-histone proteins such as p53 can also be regulated by HATs. In further studies, many other substrates of non-histone proteins were verified to be acetylated (21, 22).

Contrastingly, HDACs are a family of 18 enzymes that exhibit opposite functions to HATs. HDACs remove the acetyl group from lysine residues, which function as corepressors of transcription and reduce the acetylation level of histones. Additionally, several non-histone proteins including c-MYC, p53, and STAT3 can also be deacetylated to regulate cellular processes (23, 24). Gene silencing is caused by lysine deacetylation, which condenses chromatin and limits transcriptional activity (25). HDACs are classified into four types based on the similarity thereof to yeast proteins: Class I, II, III and IV. Class I, II and IV HDACs require the presence of Zn2^+^, while Class III HDACs (also known as sirtuins) are dependent on NAD^+^ (26). Located mainly in the nucleus, a total of four members of Class I HDACs exist: HDAC1, 2, 3, and 8, which are yeast homologs of Rpd3 (27, 28). Class II HDACs can be divided into two groups: Class IIa, which includes HDAC4, 5, 7, and 9, and Class IIb, which includes HDAC6 and 10. The two subclasses are closely related to Hda1 in yeast and can move back and forth between the cytoplasm and the nucleus, allowing Class II HDACs to regulate cytoplasmic substrates (29, 30). Homologous to Sir2 proteins in yeast, Class III HDACs can be categorized into seven elements, including sirtuin (SIRT) 1 to 7. SIRT1, 6, and 7 reside in the nucleus to regulate gene expression by deacetylating substrates (31). SIRT2 has been found to control the cell cycle in both cytoplasm and the nucleus (32, 33). Regarding SIRT3, 4, and 5, said enzymes have been reported to reside in the mitochondria to regulate the activity of oxidative stress (34). A significant characteristic of SIRTs is the ability thereof to not only deacetylate lysine residues but also act as mono-ADP-ribosyltransferases. As such, the enzymes can modulate chromatin, repair DNA and undertake other biological processes (35). In epigenetics, the single member of Class IV HDACs, HDAC11, has emerged as a particular focus of research and is located in the nucleus. Despite preliminary research showing that HDAC11 can negatively modulate IL-10, additional research is required for verification (36).

## Cancer Immunotherapeutic Molecules and the Acetylation/Deacetylation Modification Thereof

**Figure 2:**
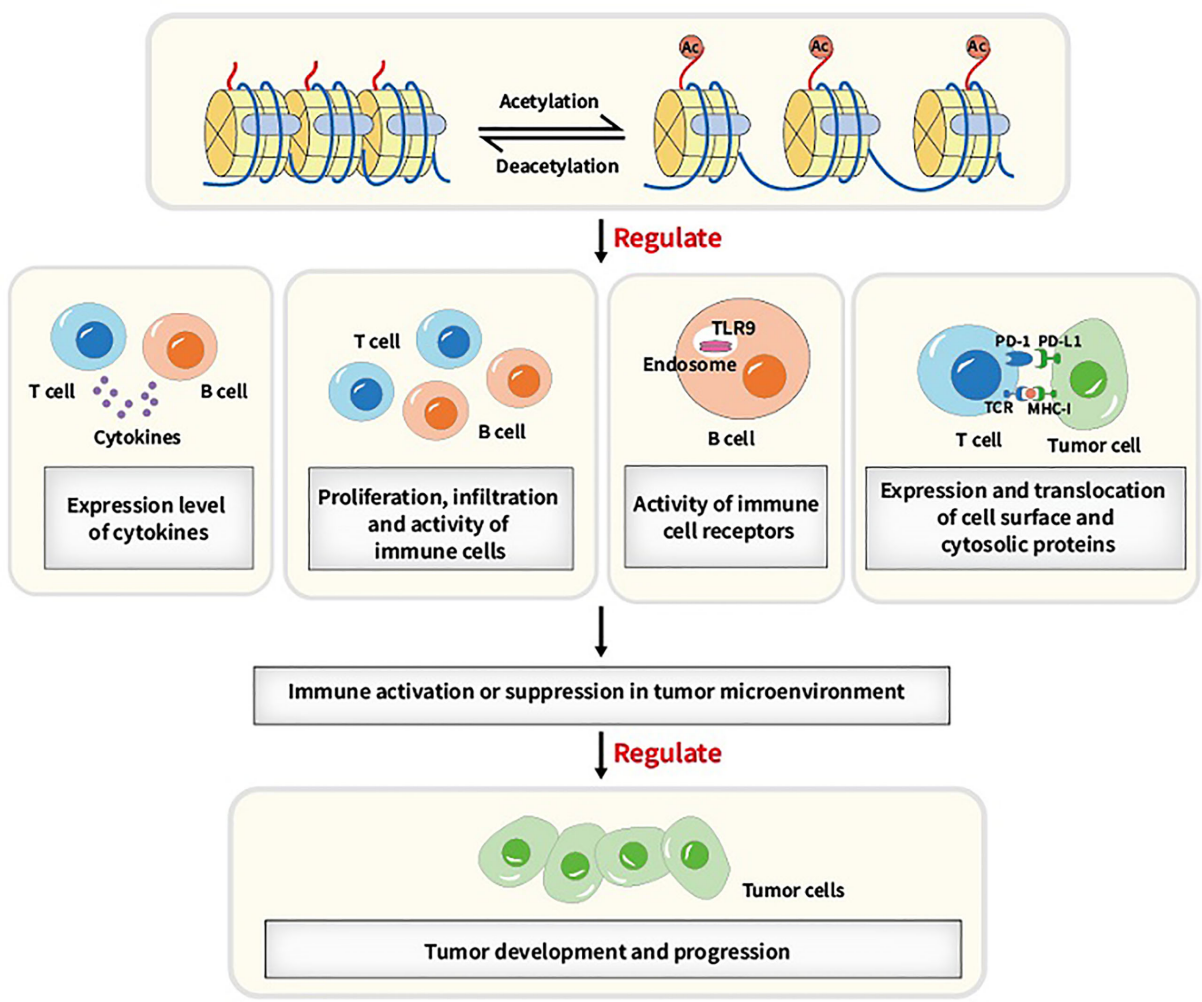
Main acetylation/deacetylation-immunity relationships. Acetylation/deacetylation process can modulate immune activity and response *via* various ways, for instance, regulating the expression level of cytokines, regulating proliferation, infiltration and activity of immune cells, regulating the activity of immune cell receptors and regulating the expression and translocation of cell surface and cytosolic proteins, which causes immune activation or suppression in tumor microenvironment and thus influencing tumor development and progression.

A number of biological processes including DNA damage repair, immunological response and cell cycle arrest require lysine acetylation/deacetylation. Abnormalities in the acetylation/deacetylation process, such as the dysregulation of the regulating enzymes, have been associated with cancer (37). In previous studies, aberrant expression levels and mutations or translocations of lysine acetylation/deacetylation regulators have been demonstrated in a variety of cancers (38). Acetylation/deacetylation process can modulate immune activity and response *via* various ways, thus influencing tumor development and progression (refer to **Figure 2**). Several specific immune-related targets of acetylation/deacetylation modification are listed as follows (refer to **Table 1**).

**Table 1:** Several specific immune-related targets of acetylation/deacetylation modification.

Molecules	Enzymes	Modification Sites	Cancer Types	Functions	References
PD-L1	p300	Lys263	–	Acetylates PD-L1 and inhibits its translocation into the nucleus.	(39)
	HDAC2	–	–	Inhibits PD-L1 acetylation mediated by p300, which increases the nuclear PD-L1 level.	(39)
	HAT1	H4K5, H4K12	Pancreatic cancer	Increases PD-L1 expression through BRD4.	(40)
	HDAC1, p300, CBP	–	Breast cancer	Modulates chromatin structure and enables transcription factor binding, which increases PD-L1 expression.	(41)
	HDAC6	–	Melanoma	Recruits and activates STAT3, which increases PD-L1 expression.	(42)
	HDAC3	H3	Non-small cell lung cancer	Decreased COP1 can cause c-Jun accumulation and suppress HDAC3 expression, which increases PD-L1 expression.	(43)
PD-1	p300, CBP, Tip60	K120, K164	Lung cancer	Facilitates PD-1 transcription and expression through acetylated p53.	(44)
CTLA-4	p300, CBP	–	Melanoma	Facilitates the transcriptional activation of CTLA-4 licensed by IFN-γ signaling.	(45)
	HDACs	H3K27	–	Regulates CTLA-4 expression.	(46)
TIM-3/LAG-3	HDAC6	–	Melanoma	HDAC6 suppression can decrease the expression level of TIM-3 and LAG-3.	(47)
B7-H3	MSL complex (including MOF)	H4K16	Nasopharyngeal carcinoma	PBK phosphorylates MSL1, promotes enrichment of MSL complex on B7-H3 promoter, leading to increased H4K16 acetylation and the activation of B7-H3 transcription.	(48)
	SIRT1	–	Colorectal carcinoma	MiR-34a can inhibit SIRT1 and consequently cause the acetylation of NF-κB subunit p65 (a-p65), thereby facilitating its binding with B7-H3 promoter and increasing the transcription and expression of B7-H3.	(49)
OX40	SIRT1, SIRT7	–	–	OX40 can induce the expression of BATF and BATF3, which bind to Foxp3 promoter and contribute to a closed chromatin configuration to suppress Foxp3 expression through the recruitment of SIRT1 and 7.	(50)
	p300, CBP, PCAF, SRC-1	H4	–	NF-κB can recruit p300, CBP, PCAF and SRC-1 to acetylate OX40 promoter on histone H4 substrate, upregulating the expression of OX40.	(51)
	HDAC1	–	–	NF-κB p50 can recruit HDAC1 to exert its deacetylation effect, which cause the down-regulation of OX40.	(51)
OX40L	HDAC1, HDAC3	–	Ovarian cancer	HDAC1 and HDAC3 can bind at OX40L promoter to downregulate OX40L expression.	(52)
4-1BBL	HDAC1, HDAC3	–	Ovarian cancer	HDAC1 and HDAC3 can bind at 4-1BBL promoter to downregulate 4-1BBL expression.	(52)
	–	–	Cutaneous T-cell lymphoma	GATA6 is epigenetically overexpressed in CTCL cells on account of DNA histone acetylation, which combines with the promoter region of 4-1BBL and thus facilitating the expression of 4-1BBL.	(53)
CD70	HDAC1	–	–	RFX1 can recruit HDAC1 to the promoter region of CD70 where it deacetylates histone substrate, causing a more condense chromatin structure and decrease expression level of CD70.	(54)
	HDAC2, HDAC7	H3	–	TSA can suppress the activity of HDAC2 and HDAC7, which causes the increased H3 acetylation at CD70 promoter and thus facilitating the expression of CD70.	(55)
	HDAC5	–	–	Overexpression of HDAC5 is related with upregulated CD70, inducing growth suppression and apoptosis of tumor cells.	(56)

HATs, histone acetyltransferases; HDACs, histone deacetylases; SIRTs, sirtuins; CBP, CREB-binding protein; CTLA-4, cytotoxic T-lymphocyte-associated antigen 4; PD-L1, programmed death-1 ligand; PD-1, programmed death-1; pSTAT1, phosphorylated STAT1; BRD4, bromodomain-containing 4; OX40, tumor necrosis factor receptor superfamily member 4; OX40L, OX40 ligand; BATF, basic leucine zipper transcription factor ATF-like; 4-1BBL, 4-1BB ligand; RFX1, regulatory factor X 1; STAT3, signal transducer and activator of transcription 3; COP1, constitutive photomorphogenesis protein 1; tim, TIM-3, T-cell immunoglobulin and mucin domain 3; LAG-3, lymphocyte-activation gene 3.

### PD-L1

The binding of programmed death-1 ligand (PD-L1, also known as B7-H1 or CD274) to programmed death-1 (PD-1) produces a large immune effect in pathological and physiological occurring situations (57). Oncogenic PD-L1 has been found to be overexpressed in many solid tumors, such as melanoma, non-small cell lung cancer, and breast cancer (58). PD-L1 is stimulated by PD-1 and signals cancer cells to maintain survival, which leads to cancer cells becoming resistant to Fas signaling pathway-induced apoptosis (59). Further, in the absence of PD-1 signaling, PD-L1 blocks the cytotoxic activity of malignant tumor cells in response to interferons (Type I and II) and CTL cells. Thus, PD-L1 is a significant factor in regulating host immune responses in the context of cancer (60). As one of the most compelling immune checkpoints, PD-L1 has become a target for clinical use, and several therapeutic agents (like PD-L1 inhibitors) have been approved for cancer immunotherapy, such as Durvalumab and Atezolizumab (61). The increased expression level of PD-L1 in immune cells has been reported to be linked with a higher response rate to ICIs and better prognosis in several cancers, including urothelial carcinoma (62), Hence, PD-L1 could be applied in effective cancer immunotherapy strategies.

A large number of PTMs types have been shown to regulate PD-L1, namely ubiquitination, phosphorylation, glycosylation, and acetylation/deacetylation (63). PD-L1 can be acetylated through p300 acetyltransferase at Lys263 in the cytoplasmic region and acetylated PD-L1 is restrained to be translocated into the nucleus. HDAC2 was also found to be capable of interacting with PD-L1 and inhibiting PD-L1 acetylation mediated by p300, which could increase the nuclear PD-L1 level and facilitate the immune surveillance evasion of tumor cells (39). Such findings provide another theoretical foundation for regulating PD-L1 by acetylation or deacetylation in cancer immunotherapy. Suppressing the shuttle of PD-L1 from cytoplasm to the nucleus by activating p300 or inhibiting the synergy of HDAC2 with PD-1 blockade may recover host immune responses. Such recovery is achieved by facilitating the infiltration of CD8^+^ T cells and downregulating the expression level of TNF-α, which may elevate the immunotherapeutic efficacy for cancer. According to another previous study, the expression of PD-L1 is also relevant to HAT1 expression in pancreatic cancer. HAT1 can acetylate H4K5 and H4K12 residues, further promoting the combination of transcriptional factor BRD4 (bromodomain-containing 4) and the PD-L1 promoter to facilitate PD-L1 transcription. Knockdown of HAT1 can upregulate tumor infiltration of immune effectors such as CD45^+^CD4^+^ and CD45^+^CD8^+^ T cells, suppress the infiltration of CD11b^+^Gr1^+^ myeloid cells in tumors and decrease the expression level of PD-L1, which may inhibit tumor proliferation and promote the efficacy of the immune checkpoint blockade (40). As such, targeting HAT1 alone or in combination with ICIs could be a novel strategy in cancer immunotherapy. Moreover, Darvin P et al. observed a consistent overexpression of HDAC1 and p300/CBP enzymes in breast cancer cells, which exhibited a close relationship with upregulated PD-L1 level and increased breast cancer aggressiveness (41). Another study also reported that HDAC6 can recruit and activate the signal transducer and activator of transcription 3 (STAT3), which increases the expression of PD-L1 through STAT3-mediated activation of the PD-L1 promoter in melanomas. By modulating various mechanisms involved in immune recognition and tumor survival, such as down-regulating PD-L1, PD-L2 and B7-H4, the application of HDAC6 inhibitors exhibited an anti-tumor effect, which might provide theoretical basis to apply HDAC6 inhibitors in cancer immunotherapy (42). In cisplatin-resistant non-small cell lung cancer cells, a decreased constitutive photomorphogenesis protein 1 (COP1) level caused the increased accumulation of c-Jun, which suppressed HDAC3 expression and facilitated acetylation of the PD-L1 promoter at the H3 site, thereby upregulating the expression of PD-L1. Increased PD-L1 was found to be crucial for inhibiting the proliferation of CD3^+^ T cells, indicating that the immune checkpoint blockade and HDAC3 activation might be effective therapeutic methods for reversing T cell-related immune suppression in drug-resistant tumors (43). The aforementioned studies reflect the indispensable role of HDACs in PD-L1 regulation, verifying the rationality of the extensive application of HDACi in clinical practice.

However, in the acetylation/deacetylation modification of PD-L1, there remain certain aspects that require further clarification. Although the aforementioned studies provide a macro-level explanation for the link between enzymes and PD-1/PD-L1 expression, the specific signaling pathway and regulatory mechanism of PD-1/PD-L1 modification by HATs/HDACs are still unclear. Further research concerning the association between HATs/HDACs and PD-1/PD-L1, which can clarify the upstream/downstream molecules in the signaling pathway, may provide new targets and innovative ideas for cancer immunotherapy. Further, most of the regulating pathway and molecular mechanisms of PD-L1 modification by acetylation/deacetylation have been identified at the cellular or animal model stage, and no findings have been made using clinical trials. To expand cancer immunotherapy strategies, further clinical studies that confirm the effect of PD-L1 acetylation/deacetylation modification on tumor development and progression are crucial.

### PD-1

PD-1 has been validated to maintain immune homeostasis through PD-1/PD-L1 interactions and is also involved in chronic infections and malignancies. Wherry et al. reported that T cells showed a dysfunctional state and lost the immune function thereof in a mouse model infected with lymphocytic choriomeningitis virus (LCMV). Notably, the expression level of PD-1 was found to be upregulated, a result that can be considered as one of the characteristics of dysfunctional T cells (64). In human malignancies, T cells with upregulated PD-1 are corelated with tumor reactivity, which is linked with PD-1 expression driven by antigens in both tumors and peripheral blood (65). In hepatocellular carcinoma cells, researchers have demonstrated that blocking the interaction between PD-1 and PD-L1 can suppress the immunosuppressive activity of B cells with high PD-1 level (66). Through reactivating T cell function and restoring host immune responses, several effective antibodies against PD-1 that block PD-1/PD-L1 interaction have been developed and applied in cancer immunotherapy (58).

One study showed that PD-1 can be modified by acetylation. The transcription and expression level of PD-1 can be upregulated by acetylated p53, which is modified by p300, CBP and Tip60 acetyltransferases at K120/K164 sites. After acetylation by the aforementioned HATs, the acetylated non-histone protein p53 can further recruit the acetyltransferase cofactors to interact with the PD-1 promoter, which facilitates the acetylation level of local chromatin and contributes to a looser chromatin structure, thereby increasing the transcription and expression of PD-1. Upregulated intrinsic PD-1 in lung cancer cells was also found to significantly inhibit tumor growth. Using HDACi upon p53 activation might be considered as a potential strategy for cancer therapy *via* upregulation of cancer cell-intrinsic PD-1 (44). However, the aforementioned PD-1 regulatory signaling pathway in tumor suppression is regulated in an immunity-independent manner (through inhibiting the AKT/mTOR pathway). Whether tumor growth is inhibited by modulating immunological functions, such as regulating T cell activation and exhaustion, needs further experimental confirmation. Additionally, there is a scarcity of relevant studies on the acetylation/deacetylation modification of PD-1 compared with studies on PD-L1. Further studies on the signaling pathways and mechanisms of acetylation/deacetylation regulation of PD-1 may provide new therapeutic strategies and acting targets for future cancer immunotherapy.

### CTLA-4

Cytotoxic T lymphocyte-associated antigen 4 (CTLA-4, CD152 for short) belongs to the CD28 immunoglobulin subfamily and is generally considered to function as a suppressor receptor. CTLA-4 is involved in a number of suppressive activities such as inhibition of cell cycle progression, T cell proliferation, cytokine expression, and other activities, mainly through the expression of activated effector T cells (67). Further, CTLA-4 directly regulates the homeostasis of Tregs. As a recognized checkpoint in the immune system, CTLA-4 has been extensively investigated as an immunotherapeutic target for a variety of malignancies, including melanoma (68), prostate cancer (69), and hepatocellular cancer (70). Targeting the CTLA-4 signaling pathway by antibodies or fusion proteins has attracted significant attention in the field of cancer therapy. In mouse cancer models, researchers identified that anti-CTLA-4 treatment strategies could cause tumor regression and lead to resistance to reinoculation of malignant cells (71). Based on the efficacy of blocking CTLA-4 in mouse models, several drug antibodies against CTLA-4 have been used clinically and tested in trials for advanced cancers (72).

The transcription and expression level of CTLA-4 are closely associated with the acetylation process. In prior research, histone acetylation mediated by p300/CBP was found to be critical for the transcriptional activation of CTLA-4 licensed by interferon-gamma (IFN-γ) signaling in melanoma cells. IFN-γ-regulated STAT1 can be phosphorylated *via* JAK1/2 dependent pathway and bind to the specific binding site of the CTLA-4 promoter, facilitating the acetylation process regulated by p300/CBP and increasing the expression of CTLA-4 (45). *Via* inhibiting T cell cytotoxicity and facilitating melanomagenesis and progression in melanocytes and melanoma cells, upregulated CTLA-4 induced by IFN-γ was found to contribute to an immunosuppressive microenvironment. Therefore, regulating CTLA-4 acetylation modification in combination with an anti-CTLA-4 strategy might relieve T cell deactivation and produce a curative effect in cancer immunotherapy. A further study demonstrated that low-dose HDAC inhibitor can modulate CTLA-4 expression to facilitate the natural production of Foxp3^+^ Treg cells and restore the suppressive function of Treg cells *via* the regulation of histone H3K27 acetylation in immune thrombocytopenia (ITP). Such findings indirectly reflect that HDACs are also related to CTLA-4 regulation, and that targeting HDACs to modulate the immune response may have potential application value in cancer immunotherapy (46). Despite the aforementioned findings, the specific types of HDAC that participate in such process need to be identified in further studies. In addition to studies on the acetylation/deacetylation of PD-1/PD-L1, there is also a scarcity of studies on the detailed mechanism and specific signaling pathways of CTLA-4 expression regulated by HATs or HDACs. Further studies will be meaningful for cancer immunotherapy, and carefully designed experiments are needed in the future.

### TIM-3/LAG-3

As members of the immune checkpoint family, T-cell immunoglobulin and mucin domain 3 (TIM-3) and lymphocyte activating gene-3 (LAG-3) have negative immunomodulatory functions in tumors. In terms of TIM-3 and LAG-3, the mechanisms of action and potential as therapeutic targets have been validated to a certain extent (73). In particular, TIM-3 expression could be found on functionally exhausted T cells in several mouse tumor models and in a large number of cancer cells such as melanoma and non-small cell lung cancer (74, 75). The levels of TIM-3 have been associated with advanced tumor nodal metastasis (TNM) and poor prognosis and can therefore be considered as a negative prognostic biomarker for different types of cancer (76). Regarding LAG-3, which is upregulated in activated T cells and NK cells, said gene can cause T cell dysfunction or apoptosis. Such result may be a protective mechanism adopted by tumor cells to escape elimination by the immune system (77). LAG-3 has been reported to be co-expressed with TIM-3 and, most typically, PD-1. Targeting LAG-3/TIM-3 alone or through a combination of drugs acting on different immune checkpoints may be effective (78).

A previous study reported that suppressing HDAC6 can decrease the expression level of LAG-3, TIM-3 and PD-1, relieve the suppressive function of Treg cells and upregulate TIL cytolytic function in melanoma patients, potentially indicating that HDAC6 is involved in the regulatory pathways of LAG-3 and TIM-3. A further indication is that blocking HDAC6 activity may be effective in alleviating T cell suppression. Selective inhibitors of HDAC6 enhance T cell immune properties in melanoma patients, providing a theoretical basis for further studies to evaluate the potential clinical efficacy thereof (47). However, there are limited researches on the acetylation/deacetylation modulation of TIM-3 and LAG-3 compared with researches on PD-L1. Further studies on whether TIM-3 and LAG-3 are regulated by other acetylation/deacetylation molecules and the specific signaling pathways may provide novel targets and therapeutic strategies for future cancer immunotherapy.

### B7-H3

As a recently discovered member of the B7 family, B7-H3 (also known as CD276) exhibits a dual role in immune cell responses and has been found to be upregulated in a variety of cancer types, such as colorectal and breast cancers (79, 80). B7-H3 has been reported to have a significant co-stimulatory molecular function in various immune cell responses, such as IFN-γ production and T cell activation (81). In a previous study, the expression level of the costimulatory molecule B7-H3 was found to be upregulated in patients with pancreatic cancer, being associated with improved treatment outcome and better postoperative prognosis (82). In patients with colon adenocarcinoma, B7-H3 also exhibited antitumor effects and could be considered as a potential therapeutic molecule in cancer therapy (83). At the same time, B7-H3 also has the ability to inhibit T cell proliferation. Upregulation of B7-H3 is positively associated with poor clinical outcomes in patients with oral squamous cell carcinoma (OSCC). Notably, the expression of B7-H3 promotes tumor growth, while the blockade of B7-H3 inhibits tumor progression (84).

A recent study elucidated a potential upregulation mechanism of B7-H3 in nasopharyngeal carcinoma (NPC). PDZ-binding kinase (PBK) phosphorylates MSL1 and facilitates the enrichment of the MSL complex (consisting of MSL1 and MSL2, MSL3, and MOF) on the B7-H3 promoter region, thereby contributing to the acetylation thereof at H4K16 residue and increasing the transcription of B7-H3. High expression of B7-H3 is positively correlated with CD8-naive, Th2 and nTreg cells but is negatively correlated with Th1, Tfh and CD4 T cells. The cytotoxic T-cell function that attacks NPC cells is also suppressed by increased B7-H3 (48). Thus, targeting both B7-H3 and the acetylation signaling pathway may provide therapeutic benefits for NPC patients. Moreover, another study reported a new signaling pathway through which miR-34a upregulates B7-H3, providing a theoretical basis for targeting B7-H3 in cancer immunotherapy. B7-H3 can be upregulated through the miR-34a/SIRT1/NF-κB/B7-H3 axis in colorectal carcinoma. MiR-34a inhibits SIRT1, which causes the acetylation of the NF-κB subunit p65 (a-p65), thereby promoting the binding thereof to the B7-H3 promoter and increasing the transcription and expression of B7-H3. Upregulated B7-H3 promotes an abundant release of pro-inflammatory cytokines, including TNF-α, IFN-γ, IL-2, IL-6 and IL-10, resulting in the inhibition of the antitumor immune response and the subsequent promotion of tumor growth (49). Therefore, targeting B7-H3 related molecules in acetylation/deacetylation signaling pathways may affect the expression level of B7-H3, which is a significant factor in regulating the immune response in the tumor microenvironment. However, due to dual effect of B7-H4 in the cellular immune response, further research is needed to explore whether modulating the acetylation/deacetylation process of B7-H4 can produce a specific therapeutic effect in cancer immunotherapy.

### OX40/OX40L

Tumor necrosis factor receptor superfamily member 4 (OX40, also known as CD134) and the OX40 ligand (OX40L, also known as CD134L or CD252) thereof are indispensable immune checkpoints that have been reported to be expressed on various cell types, such as activated T cells, B cells, and endothelial cells (85). OX40/OX40L interaction promotes T cell proliferation, upregulates cytokine production, and suppresses the immunosuppressive effects of Treg cells, thereby promoting immune responses (86). In a number of studies, OX40 was found to be expressed on tumor infiltrating lymphocytes (TILs) in many types of cancers, namely colorectal, gastric and ovarian, thereby regulating the development and progression of cancer (87–89). The combination of OX40 agonists with PD-1 blockade has been revealed to be effective in cancer immunotherapy, being able to inhibit the growth of ovarian tumor cells (89). The combination of anti-OX40 therapy with cytokines or radiotherapy can also exert antitumor effects in cancer immunotherapy (90, 91).

OX40 and OX40L have been found to be involved in acetylation/deacetylation regulatory signaling pathways. In a previous study, OX40 could induce the expression of basic leucine zipper transcription factor ATF-like (BATF) and BATF3, both of which bind to the Foxp3 promoter and contribute to a closed chromatin configuration to suppress Foxp3 expression through the recruitment of SIRT1 and 7. In this way, the induction of iTregs was suppressed and immune suppression was relieved (50). Moreover, NF-κB can recruit p300, CBP, PCAF and SRC-1 to acetylate the OX40 promoter on the histone H4 substrate, upregulating the expression of OX40. NF-κB p50 can also recruit HDAC1 to exert a deacetylation effect, which causes the down-regulation of OX40. The differentiation of Tregs could be attributed to the chromatin structure of the OX40 promoter and the OX40 level. Hence, targeting the acetylation/deacetylation process that regulates OX40 expression might affect the Tregs level and the host immune response (51). Another study demonstrated that HDAC1 and HDAC3 can bind at the OX40L promoter to downregulate OX40L expression in chemoresistant ovarian cancer cells, which could potentially induce the immunosuppression of cancer cells to escape from immune responses (52). Therefore, targeting OX40/OX40L or the upstream/downstream molecules thereof in acetylation/deacetylation signaling pathways may provide more possibilities and better treatment efficacy for cancer immunotherapy, especially for chemoresistant tumors.

### 4-1BBL

As a member of the superfamily tumor necrosis factor (TNF), the 4-1BB ligand (known as CD137L) is expressed on activated B cells, T cells, macrophages and dendritic cells (92). By interacting with the high affinity receptor 4-1BB (CD137), 4-1BBL acts as an immunostimulant molecule to supply costimulatory messages to CD4^+^ and CD8^+^ T cells through activating the NF-κB and c-Jun signaling pathways, which modulates immune response and activity (93). Combined application of vaccine that contains RM-1 cells which expressed 4-1BBL with CTLA-4 blockade can induce the regression of prostate cancer cells, showing the treatment potency of 4-1BBL in cancer immunotherapy (69).

4-1BBL can be modulated by the acetylation/deacetylation process and is related to cancer. In a previous study, HDAC1 and HDAC3 were found to bind at the 4-1BBL promoter to downregulate 4-1BBL expression in chemoresistant ovarian cancer cells, resulting in immune escape and suppression (52). However, the expression level of 4-1BBL has diverse effects on tumor progression in different types of cancers. GATA6 has been reported to be epigenetically overexpressed in cutaneous T-cell lymphoma (CTCL) cells owing to DNA histone acetylation, which combines with the promoter region of 4-1BBL, thereby facilitating the expression of 4-1BBL and promoting tumor progression (53). Blocking 4-1BBL may provide a new immunotherapeutic strategy for the treatment of certain malignancies, despite weakening the antitumor immune response of NK cells and CD8^+^ T cells. Hence, the expression level of 4-1BBL in different types of cancers and the influence thereof on tumor progression need to be further clarified through experiments, so as to provide a theoretical basis for cancer immunotherapeutic strategies. In cancer immunotherapy, 4-1BBL has been utilized according to the immunological characteristics thereof. To date, whole-cell vaccines with 4-1BBL cDNA modified tumor cells have been reported as the most common immunotherapeutic application of 4-1BBL. Researchers have found that the transfer of 4-1BBL cDNA into a mouse squamous cell carcinoma effectively activated primary anti-tumor immune responses of CD8^+^ T cells (94). The combined application of acetylation/deacetylation modulation and tumor cell vaccines may provide a new direction for cancer immunotherapy and needs to be validated by further research.

### CD70

CD70 (also referred to as TNFSF7) is a Type II transmembrane glycoprotein and belongs to the TNF superfamily (95). The interaction between CD70 and the receptor CD27 has been demonstrated to facilitate cytotoxic T cell responses and induce the cytokine production of CD4^+^ and CD8^+^ T cells, thereby regulating the cellular immune response (96). Upregulated CD70 expression has been observed in various types of malignancies, being linked with poor clinical outcomes and prognosis in certain cancers such as breast cancer and B cell lymphoma (97, 98). The aforementioned results may be explained by immune inhibition and escape resulting from an increased amount of iTregs, upregulated lymphocytes apoptosis and induced T cell exhaustion by CD70/CD27 interaction (99, 100).

In systemic lupus erythematosus (SLE), regulatory factor X 1 (RFX1) was found to be able to recruit HDAC1 to the promoter region of CD70 and deacetylate the histone substrate, resulting in a more condense chromatin structure and a decrease in the expression level of CD70. At the same time, there was a reduction in T cell activation and IgG produced by B cells (54). Moreover, TSA (a histone deacetylase inhibitor) can suppress the activity of HDAC2 and HDAC7, which leads to increased H3 acetylation at the CD70 promoter, and thus, facilitates the expression of CD70 (55). The potential rationality of using HDACi in cancer immunotherapy was also revealed, in addition to a potential direction for further clinical trials. Another study showed that overexpression of HDAC5 is related to upregulated CD70, which induces the growth suppression and apoptosis of tumor cells, indicating that targeting HDAC5 may regulate the immune response and affect tumor progression in cancer immunotherapy (56). However, most of the messages concerning CD70 acetylation/deacetylation modulation have been reported in the context of SLE. Further studies on the acetylation/deacetylation of CD70 in various tumors will be beneficial in establishing a solid theoretical foundation for cancer immunotherapy.

## Combined Application of HDACi and Other Immunotherapeutic Strategies

In recent decades, many scholars have found that epigenetic abnormalities, especially dysregulated acetylation or deacetylation, are significant features of various cancers (17). As biological inhibitors that target HDACs that are critical in deacetylation modification, HDACi are used as epigenetic regulatory agents in cancer immunotherapy. By interfering with the activity of HDAC, histone acetylation is indirectly induced to regulate gene expression and modulate host antitumor immune responses. Several studies have shown that HDACi can re-express inactivate regulatory genes in cancer cells and reverse malignant phenotypes (101). By decreasing the levels of FoxP3 and Treg, increasing the cytotoxicity of NK and CD8^+^T cells, and increasing the production of proinflammatory cytokines, HDAC inhibitors are conducive to reducing tumor formation and boosting an efficient immune response against cancer cells (102, 103). At the same time, the normal function of the immune system may be further influenced by HDAC inhibition, which can affect the expression of immune checkpoints and associated ligands. In an SM1 murine melanoma model, the HDAC6 inhibitor greatly reduced PD-L1 and PD-L2 overexpression, which increased the overall survival rate (104). As revealed in another study, HDAC6 inhibition could suppress the T cell expression levels of PD-1, TIM-3 and LAG-3, relieving T cell suppression in melanoma patients (47). The combined application of HDACi and other immunotherapeutic strategies have recently emerged as significant areas of focus in the field of tumor therapy. Several synergistic immunotherapeutic strategies are listed as follows.

## Combined Application of HDACi and ICIs

Immune checkpoints can effectively suppress immune responses or enable cancers to avoid damage from the entire immune system by activating negative regulatory pathways. Two such immune checkpoints, PD-1/PD-L1 and CTLA-4, have so far received the most attention in cancer immunotherapy. ICIs have made breakthrough progress in many cancer therapeutic fields, bringing new treatment hope to patients. Despite such progress, some patients who use ICIs exhibit no response or even develop drug resistance (105). Studies have shown that HDACi can significantly improve the immune response following the application of ICIs, and can enhance immunity to the effects of treatment. Acetylase inhibitors induce the infiltration of effector T cells, which secrete a variety of immune-related cytokines along with natural killer cells to form a critical line of defense against cancer in the body. HDACi in combination with ICIs have been shown to significantly increase the level of natural killer cells in the tumor microenvironment, resulting in a gradual decrease in tumor cell size (106). Thus, through combined application, HDACi can provide a double guarantee for the efficacy of ICIs by regulating the positive and negative mechanisms of immune response.

Many pre-clinical studies have reported the potential of combining HDACi with ICIs in cancer immunotherapy (refer to **Table 2**). For instance, HDACi in combination with anti-PD-1 or anti-CTLA-4 antibodies were found to promote the production of granase B in activated CD8^+^ T responder cells, significantly improving the infiltration and function of innate immune cells and enhancing adaptive immune response. Using a hepatocellular carcinoma preclinical model, selective HDAC8 inhibition was found to exert effective responses to anti-PD-L1 treatment by reactivating the production of T cell-trafficking chemokines (107). CG-745, a member of HDACi, has been validated to modulate the immune microenvironment and enhance the therapeutic effect of anti-PD-1 ICIs, which indicates the effectiveness of the combined application of HDACi and ICIs (108). In previous studies, the expression level of PD-L1 was increased by HDAC inhibitors, increasing the susceptibility of the host immune system to anti-PD-L1 and anti-PD-1 therapy, and promoting therapeutic results in patients (109). Combining Nexturastat (HDAC6 inhibitor) with an anti-PD-1 agent has been reported to suppress tumor growth, decrease the level of pro-tumorigenic M2 macrophages and upregulate effector T cells in melanoma (104). Moreover, the Class I HDAC inhibitor Etinostat facilitated the positive antitumor effect of PD-1 inhibition in syngeneic mouse models of lung and renal cell carcinoma, which could be attributed to the inhibition of the immunosuppressive functions of myeloid-derived suppressor cells (MDSCs) (110). In a recent study, HDACi Romidepsin was found to upregulate PD-L1 expression and the combined application of Romidepsin and anti-PD-1 antibody could enhance the antitumor therapeutic effect and partially reverse the influence of Romidepsin on CD4^+^ and CD8^+^ T cells in colon cancer (111). Further, the combined application of HDACi and antibodies against CTLA-4 could facilitate CD4^+^T cell infiltration and exert a synergistic effect on cancer treatment by intensifying anticancer immune responses (106).

**Table 2:** Combined application of HDACi and ICIs in pre-clinical studies and clinical trials.

HADCi	Combined ICIs	Phases	NCT Numbers	Cancer Types	Effects	References
PCI-34051	PD-L1 antibody	Pre-clinical	–	Hepatocellular carcinoma	Reactivating T cell-trafficking chemokines production.	(107)
CG-745	PD-1 antibody	Pre-clinical	–	–	Modulating the immune microenvironment and enhancing the therapeutic effect of anti-PD-1 ICIs	(108)
Romidepsin	PD-1 antibody	Pre-clinical	–	Lung adenocarcinoma	Increasing the susceptibility of the host immune system to anti-PD-L1 and anti-PD-1 therapy.	(109)
Nexturastat	PD-1 antibody	Pre-clinical	–	Melanoma	Suppressing tumor growth, decreasing pro-tumorigenic M2 macrophages level and upregulating effector T cells.	(104)
Etinostat	PD-1 inhibition	Pre-clinical	–	Lung and renal cell carcinoma	Facilitating the positive antitumor effect of PD-1 inhibition by inhibiting the immunosuppressive functions of MDSCs.	(110)
Romidepsin	PD-1 antibody	Pre-clinical	–	Colon cancer	Enhancing the antitumor therapeutic effect and partially reversing the influence of romidepsin on CD4^+^ and CD8^+^ T cells.	(111)
TSA	CTLA-4 antibody	Pre-clinical	–	Melanoma	Facilitating CD4^+^T cell infiltration and intensifying anticancer immune responses.	(106)
Vorinostat	Pembrolizumab	Clinical phase I/Ib	02638090	Non-small cell lung cancer	ORR = 13%	(112)
Vorinostat	Pembrolizumab	Clinical phase II	02538510	Head and neck squamous cell carcinomas and salivary gland cancer	ORR = 32%	(113)
Vorinostat	Pembrolizumab	Clinical phase II	02395627	Estrogen receptor-positive breast cancer	ORR = 4%, CBR = 19%	(114)
Entinostat	Atezolizumab	Clinical phase Ib/II	03280563	MORPHEUS receptor-positive breast cancer	ORR = 6.7%	(115)
Entinostat	Atezolizumab	Clinical phase II	02708680	Advanced triple-negative breast cancer	ORR = 10%, CBR = 37.5%	(116)
Entinostat	Atezolizumab	Clinical phase I	03024437	Renal cell carcinoma	ORR = 20%	(117)
Entinostat	Nivolumab and ipilimumab	Clinical phase I	02453620	Advanced solid tumors	ORR = 16%	(118)
Entinostat	Pembrolizumab	Clinical phase II	02437136	Non-small cell lung cancer	ORR = 9.2%	(119)
Mocetinostat	Nivolumab and ipilimumab	Clinical phase I	03565406	Melanoma	ORR = 70%	(120)
Domatinostat	Avelumab	Clinical phase II	03812796	Oesophagogastric and colorectal cancers	SD = 46.2%	(121)
Domatinostat	Pembrolizumab	Clinical phase Ib	03278665	Melanoma	CBR= 30%	(122)
Romidepsin	Nivolumab	Clinical phase I/II	02393794	Triple negative breast cancer	ORR = 44%	(123)

HADCi, histone deacetylase inhibitor; ICIs, immune checkpoint inhibitors; PD-L1, programmed death-1 ligand; PD-1, programmed death-1; MDSCs, myeloid-derived suppressor cells; CTLA-4, cytotoxic T-lymphocyte-associated antigen 4; ORR, overall response rate; CBR, clinical benefit rate; SD, stable disease.

To further investigate the efficacy of the combined application of HDACi and ICIs, researchers have recently conducted several clinical trials and made progress to a certain degree (refer to **Table 2**). For instance, in metastatic uveal melanoma, the combined application of HDACi Entinostat and PD-1 inhibitor Pembrolizumab has been validated to exert durable responses of tumor regression and prolonged survival in a phase 2 clinical trial, especially for patients with low tumor burden and *BAP1* wildtype tumors (124). Further, in a phase 1/1b study, synergizing Vorinostat with Pembrolizumab was well tolerated and exhibited a preliminary anti-tumor effect in advanced/metastatic non-small cell lung cancer (112). The combined application of Tamoxifen, Vorinostat and Pembrolizumab in a phase 2 trial exhibited clinical benefits in terms of the upregulation of exhausted CD8^+^ T cells and the depletion of Tregs, which was linked with better clinical response and prolonged PFS in ER-positive breast cancer patients. Said combination was also well-tolerated without excess toxicity (114). However, in another phase 2 trial involving the combined application of Pembrolizumab and Vorinostat, significant efficacy was reported with a relatively higher grade of toxicity compared with using anti-PD-1 monoclonal antibodies alone in recurrent metastatic head and neck squamous cell carcinomas and salivary gland cancer (113). As such, immunotherapy involving the coadministration of ICIs and HDACi may be essential in prospective cancer treatment. Such immunotherapy may be particularly beneficial for cancer patients who develop drug resistance to immune checkpoint inhibitors. With the in-depth research on the role of HDACi and ICIs in anti-tumor treatment, the synergistic application of HDACi and ICIs may emerge in the broader field of cancer research and eventually in clinical cancer immunotherapy.

## Combined Application of HDACi and Cancer Vaccine

Cancer vaccines can be applied in patients with malignant tumors to overcome the immune suppression caused by tumors and activate a patient’s immune system, so as to achieve the purpose of controlling or eliminating tumors (125). As an example, the emergence of the therapeutic HPV DNA vaccine has successfully opened up a new direction for the prevention and treatment of cervical cancer, but the antigenicity of the DNA vaccine itself is weak, and the use of DNA vaccine alone does not have a good anti-tumor effect. The decrease or deletion of MHC Class I molecule expression in cervical cancer cells makes tumor cells unable to recognize and kill CD8^+^T cells, resulting in immune escape. Hence, enhancing the expression of MHC Class I molecules in cervical cancer cells is a significant strategy for cervical cancer immunotherapy. Studies have shown that by increasing the expression of proteins encoded by the DNA vaccine, the combination of HDACi AR-42 and the CRT/E7 DNA vaccine can up-regulate the expression of MHC Class I molecules. Moreover, the expression of MHC Class I molecules can also activate the immune response of CD8^+^T cells, thereby producing effective anti-tumor effects (126).

Using MC38-CEA colon and 4T1 triple-negative breast murine carcinoma models, the combined application of Entinostat (HDACi), N-803 (IL-15 superagonist) and cancer vaccine was found to promote an anticancer effect by facilitating the infiltration of activated CD8^+^T cells and activating T cell responses to various tumor-associated antigens in the tumor microenvironment (127). Additionally, the synergistic inhibition of HDAC and proteins of bromodomain and extraterminal (BET) can also enhance the vaccine-induced CD8^+^T cell response and promote the therapeutic effect in melanoma treatment (128). Therefore, the combined application of HDACi and cancer vaccines may have promising prospects in clinical cancer immunotherapy (refer to **Table 3**). However, in addition to HDACi and cancer vaccines, other therapeutic agents were involved in the aforementioned studies. Further studies are needed to confirm whether applying HDACi alone with cancer vaccines can produce the same treatment effect in cancer immunotherapy.

**Table 3:** Combined application of HDACi and cancer vaccines/antitumor antibodies.

Combined application	HADCi	Combined Drugs	Cancer Types	Effects	References
HDACi Cancer vaccines	AR-42	CRT/E7 DNA vaccine	Cervical cancer	Up-regulating the expression of MHC class I molecules and activates the immune response of CD8^+^T cells.	(126)
	Entinostat	Cancer vaccine, N-803	Colon and breast carcinoma	Promoting the anticancer effect by facilitating infiltration of activated CD8^+^T cells and activating T cell responses to various tumor-associated antigens in tumor microenvironment.	(127)
	Romidepsin	Cancer vaccine, IBET151	Melanoma	Enhancing vaccine-induced CD8^+^T cell response.	(128)
HDACi Antitumor antibodies	SNDX-275	Trastuzumab	Breast cancer	Promoting Trastuzumab-induced growth inhibition by blocking the Akt signaling pathway and increasing DNA breakage.	(129)
	Chidamide	Rituximab	Diffuse large B-cell lymphoma	Upregulating CD20 and inhibiting the growth of diffuse large B-cell lymphoma cells.	(130)
	Entinostat	Rituximab	Lymphoma	Promoting the anticancer effects.	(131)
	Vorinostat	Rituximab	Lymphoma	Enhancing cell cycle arrest and restoring the sensitivity of Rituximab-resistant lymphoma cells to chemotherapeutic drugs.	(132)

## Combined Application of HDACi and Antitumor Antibodies

Therapeutic antibodies are laboratory-made antibodies designed to destroy tumor cells in different ways, including antibody-dependent cell-mediated cytotoxicity (ADCC), complement-dependent cytotoxicity (CDC), and direct induction of apoptosis by antibodies (133). Several therapeutic antibodies have been approved for the clinical treatment of cancer. In 1997, Rituximab (Rituxan), the first chimeric antibody against CD20, was approved by the FDA for the treatment of non-Hodgkin’s lymphoma. Trastuzumab (Herceptin), the first humanized anti-HER2 monoclonal antibody, was marketed in 1998 for the treatment of breast cancer. Several other directly targeted antitumor antibodies, such as Panitumumab, Cetuximab, and Adesimab, have since received FDA approval for anticancer effects in cancer immunotherapy (134–136).

The combination of HDACi and antitumor antibodies has been reported to enhance antitumor responses and promote the therapeutic efficacy of cancer immunotherapy in numerous studies. By blocking the Akt signaling pathway and increasing DNA breakage, the HDAC Class I inhibitor sNDX-275 can significantly promote Trastuzumab-induced growth inhibition in erbB2-overexpressed breast cancer cells, which may also overcome Trastuzumab resistance in breast cancer immunotherapy (129). Treatment with the HDAC inhibitor Chidamide in synergy with Rituximab was found to significantly upregulate CD20 and inhibit the growth of diffuse large B-cell lymphoma (DLBCL) *in vitro* and *in vivo*, confirming that Chidamide may have application prospects in DLBCB immunotherapy (130). A similar study confirmed the effectiveness of the combination of HDACi and antitumor antibodies, in which Entinostat was reported to promote the anticancer effects of Rituximab and chemotherapeutic agents in B-cell lymphoma (131). Another HDACi, Vorinostat, enhanced cell cycle arrest and restored the sensitivity of Rituximab-resistant lymphoma cells to chemotherapeutic drugs (132). Thus, the synergistic application of HDACi and antitumor antibodies may provide a new direction for improving the effectiveness of immunotherapy in cancer treatment (refer to **Table 3**).

## Discussion

As a significant epigenetic regulatory mechanism, acetylation/deacetylation has been found to well regulate the process of gene expression transcription and modulate the host immune response in various cancers, further aiding tumor development and progression by regulating the expression of oncogenes or tumor suppressor genes and weakening the anti-tumor immune response (137). Numerous studies have reported that targeting HATs and HDACs that are essential in the acetylation/deacetylation process can modulate epigenetic signaling pathways and recover the normal expression level of numerous cancer-related genes and anti-tumor immune responses, thereby offering treatment benefits in cancer immunotherapy (138).

Among the various substances that can target HATs/HDACs directly, or those associated with molecules in acetylation/deacetylation-relevant signaling pathways, HDAC inhibitors have become a large focus of research and been applied with various degrees of success in different settings to treat cancer (139). Since 2006, four different types of HDAC inhibitors have been authorized by the FDA for clinical use, and over 20 different HDAC inhibitors are currently being tested in clinical studies (140). Notably, as a trending field in oncology research, the therapeutic effect of cancer immunotherapy can be elevated in combination with HDAC inhibitors, showing the significance of combining multiple therapeutic methods in the practice of cancer treatment. The efficacy of HDAC inhibitors can be promoted by means of the synergetic application of numerous immunotherapeutic approaches, including cancer vaccines, immune checkpoint inhibitors and others (141).

Many immune-related molecules are not only modified by acetylation/deacetylation, but also by other PTMs such as phosphorylation, ubiquitination, and methylation to regulate the expression level thereof and further modulate host immune responses. Examples include phosphorylated PD-L1 by glycogen synthase kinase 3β (GSK3β), which can be further degraded by ubiquitin-proteasome system and Olaparib, a PARP1 inhibitor that maintains and upregulates the expression level of PD-L1 in cancers by suppressing the activity of GSK3β, thereby inhibiting the antitumor immune response and facilitating the tumor immune escape (142). Additionally, acetylation/deacetylation can act synergistically with other types of PTMs on an immune-related molecule to regulate the expression thereof. Consistently Photomorphogenic 1 (COP1) is a ubiquitin E3 ligase, and the reduction thereof can inhibit c-Jun ubiquitination degradation, maintain the stability and facilitate phosphorylation by C-Jun N-terminal kinase (JNK), further promoting the translocation of c-Jun into the nucleus and suppressing the production of HDAC3. Such effects result in a looser chromatin structure by acetylation and increases the expression of PD-L1 (143). Hence, the improvement of anticancer immunotherapeutic efficacy may be achieved through the combination of multiple drugs acting on diverse PTMs or the application of a single chemical compound that targets various substrates. The synergistic application of drugs that modulate acetylation/deacetylation and other PTMs represents a promising treatment approach in future cancer immunotherapy.

Despite a number of researchers having reported anticancer effects in immunotherapy that targets the acetylation/deacetylation process and related molecules, there are still questions that warrant further investigation. For instance, numerous immune-related molecules that regulate antitumor immune responses have not been reported to be altered by acetylation or deacetylation. T-cell immunoglobulin and the ITIM domain (TIGIT) is an emerging immune checkpoint that suppresses innate and adaptive immunity, thereby inhibiting antitumor immune responses. The combined application of drugs that block TIGIT and PD-1 (Atezolizumab/Tiragolumab) can upregulate CD8^+^ T cell expression and function, which may be considered as a potential cancer immunotherapy strategy (144). Another immune regulatory protein, V-domain Ig suppressor of T cell activation (VISTA), can maintain quiescence on T cells and myeloid-derived cells. In a CT26 colon cancer mouse model, immunotherapy applied in combination with VISTA and PD-L1 ICIs contributed to tumor regression and led to long-term survival in the mouse model, demonstrating the rationale for targeting VISTA in cancer immunotherapy. Further studies of acetylation/deacetylation modifications of immune-related molecules, such as the aforementioned novel immune checkpoints, may facilitate broader ideas and more effective therapeutic strategies for future cancer immunotherapy. Further, many cytokines have also been found to participate in the regulation of immune homeostasis and response such as transforming growth factor (TGF)-β, IFN-γ and others. TGF-β can enhance the expansion of Tregs and suppress the function of effector T cells, causing immune evasion in tumor microenvironments and poor responses to cancer immunotherapy (145). IFN-γ is crucial for NK, NKT, and T cells trafficking into tumors, which regulates the immune response and tumor progression (146). Targeting the acetylation/deacetylation process to modulate the expression of the aforementioned critical cytokines may relieve immunosuppression and reactivate the immune response in the tumor microenvironment, thereby promoting the efficacy of cancer immunotherapy.

Regarding the existing studies on the regulation of immune-related molecules by acetylation or deacetylation, the focus has mostly been on the regulatory effect of HDACs, and there is a scarcity of studies on the modification of immune-related molecules by HATs. In the clinical practice of cancer immunotherapy, HDACi have been approved and demonstrated to be valid in cancer treatment. However, unlike HDACi, HAT inhibitors have not been applied in clinical therapy and can be regarded as being stagnant in experimental phases. CCS1477, for example, is the only CBP/p300 inhibitor currently undergoing clinical trials. Even if HAT inhibitors have an essential function in tumor suppression and may exhibit a potential immunotherapeutic effect in cancer treatment, the significance of HAT inhibitors has been well-covered by the in-depth research on HDACi. HAT inhibitors and the modification signaling pathways through which HATs regulate immune-related molecules are of equal significance and deserve the attention of more scientists.

Moreover, a further challenge that inhibits the utilization of HDAC inhibitors is the adverse effects and toxicity observed in early-phase clinical trials. Taking HDACs as an example, only 18 HDACs are responsible for modulating a large number of lysine acetylation residues in over 2000 proteins. Even though HDAC inhibitors can reactivate tumor suppressors, there will be a negative effect on other genes. Future strategies should be directed at developing selective inhibitors to target specific acting sites rather than enzymes, with the aim of overcoming drug toxicity and resistance in cancer immunotherapy.

In addition, the focus of most clinical trials has been on the combination of HDACi and ICIs, and there are fewer clinical studies on the anti-tumor efficacy of HDACi combined with cancer vaccines and antitumor antibodies. Future studies on such aspects may provide new strategies for cancer immunotherapy.

## Conclusion

**Figure 3:**
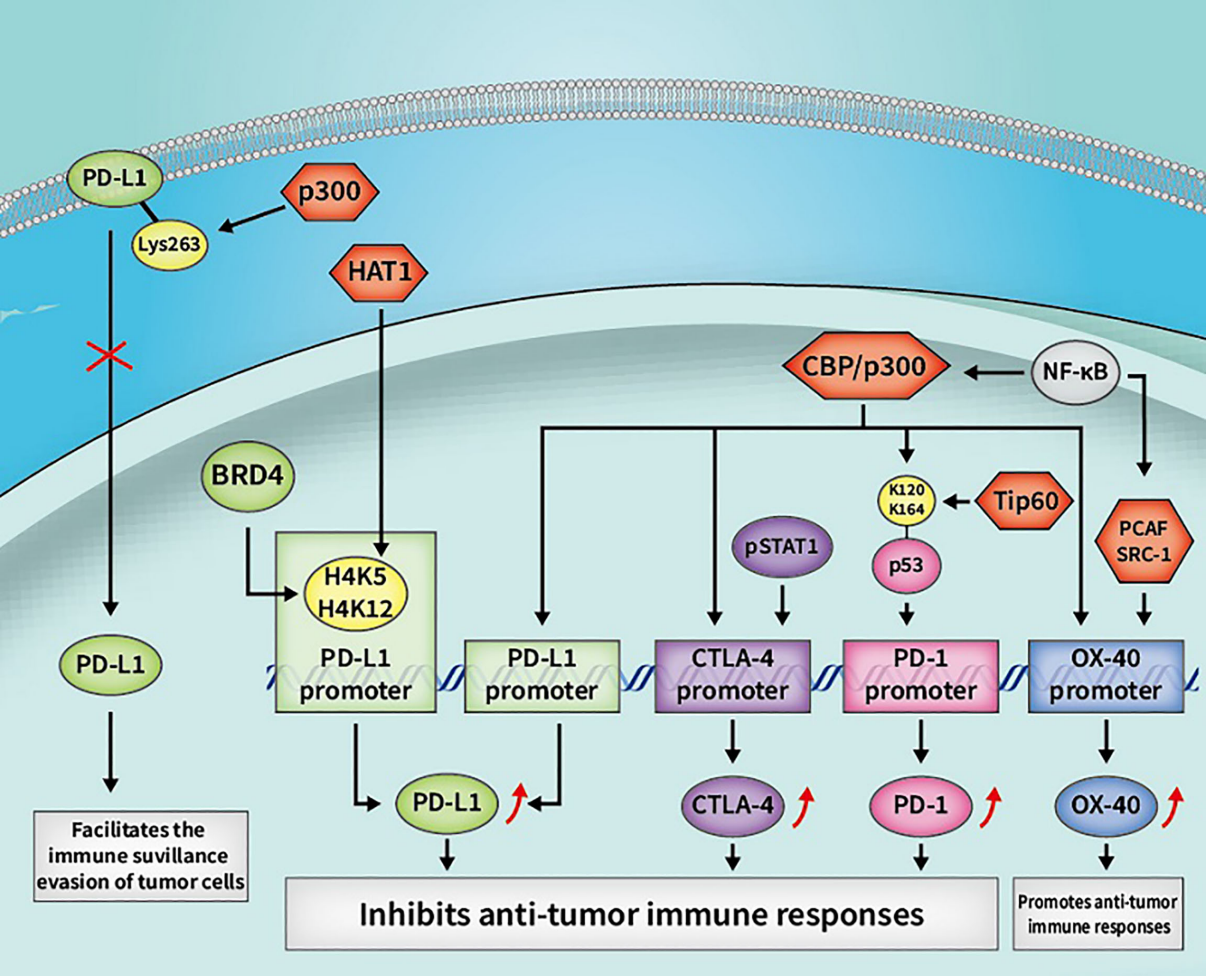
Some cancer immunotherapeutic molecules and signaling pathways regulated by HATs. HATs, histone acetyltransferases; CBP, CREB-binding protein; OX40, tumor necrosis factor receptor superfamily member 4; CTLA-4, cytotoxic T-lymphocyte-associated antigen 4; PD-L1, programmed death-1 ligand; pSTAT1, phosphorylated STAT1; BRD4, bromodomain-containing 4.

**Figure 4:**
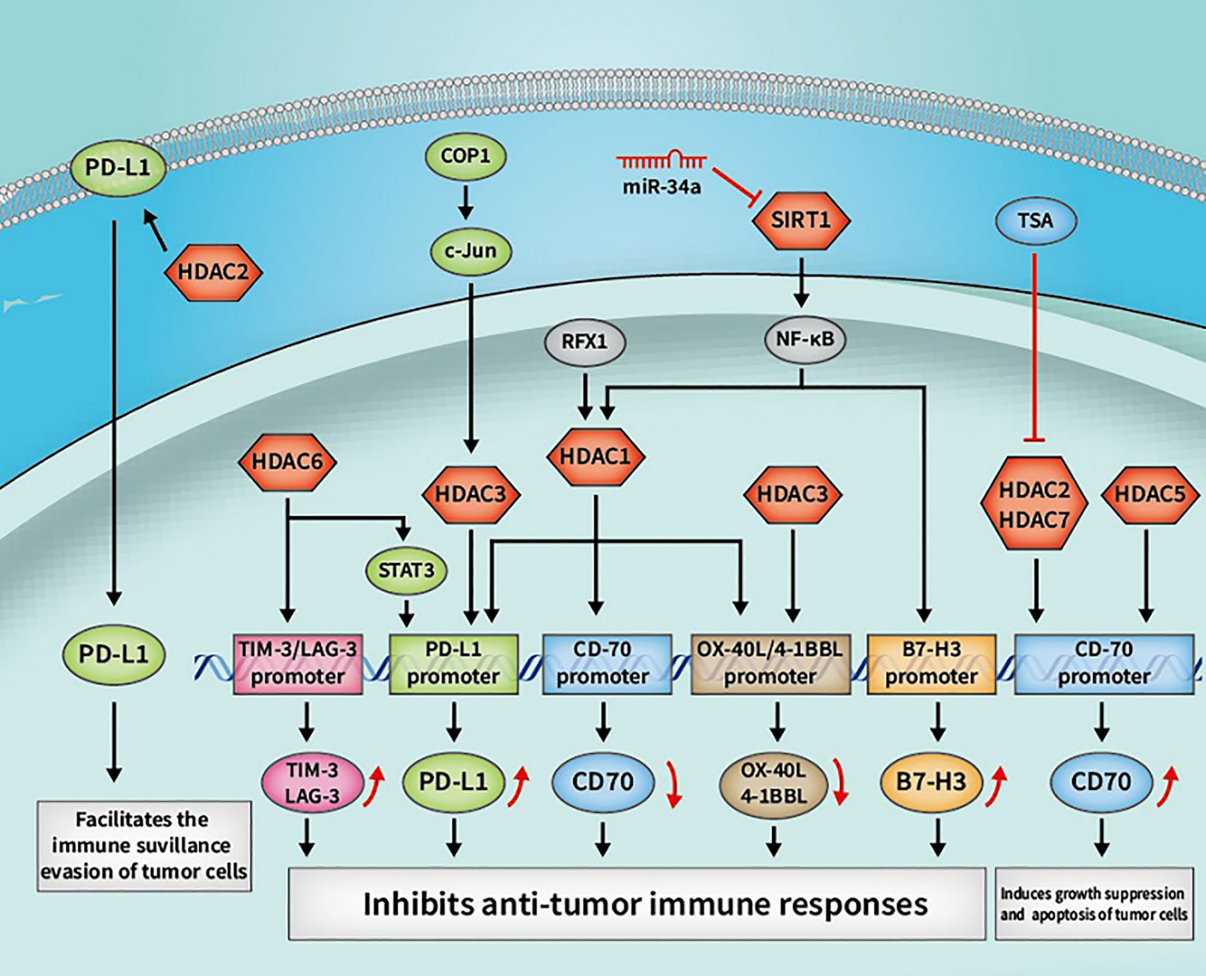
Some cancer immunotherapeutic molecules and signaling pathways regulated by HDACs. HDACs, histone deacetylases; SIRTs, sirtuins; OX40, tumor necrosis factor receptor superfamily member 4; OX40L, OX40 ligand; 4-1BBL, 4-1BB ligand; RFX1, regulatory factor X 1; PD-L1, programmed death-1 ligand; STAT3, signal transducer and activator of transcription 3; COP1, constitutive photomorphogenesis protein 1; tim, TIM-3, T-cell immunoglobulin and mucin domain 3; LAG-3, lymphocyte-activation gene 3.

In summary, as a significant PTM, lysine acetylation/deacetylation mediated by HATs/HDACs is crucial in regulating various signaling pathways, and also in modulating cancer development and progression by regulating host antitumor immune responses (refer to **Figures 3**, **4**). Owing to the indispensability thereof, lysine acetylation/deacetylation has received an increasing amount of attention from scientists. The aim of research in the relevant field is to find efficient methods for clinical cancer immunotherapy. Although targeting crucial enzymes in the acetylation/deacetylation pathway, such as HATs/HDACs, has been shown to recover immune response to exert a cancer therapeutic impact, and certain HDAC inhibitors have even been licensed by the FDA for clinical use, there are still a number of issues to be resolved. The elucidation of detailed molecules in the acetylation/deacetylation pathway, the discovery of new HAT/HDAC targeted compounds, and the combination of HAT/HDAC inhibitors with other drugs, such as immune checkpoint inhibitors, can all contribute to the finding of new targets and ideas for future cancer immunotherapy.

## Author Contributions

XY, JH, and JW designed the study. PD, ZM, DLi, MP, and HL searched the literature and wrote the manuscript. YF, YZ, CS, MJ, and DLu searched the literature and made the table, and PD draw the figures. All authors read and approved the final manuscript.

## Funding

This work was supported by the National Natural Science Foundation of China (82103508, 81871866, 82173252), Shaanxi Special Support Plan-Program for Leading Talents of Science and Technology Innovation (No. 2019 Special Support Plan), the Natural Science Foundation of Shaanxi Province (2016SF-308, 2019SF-033, 2021SF-158, 2022SF-145) and Project of Tangdu Hospital, the Air Force Military Medical University (No. 2018 Key Talents).

## Conflict of Interest

The authors declare that the research was conducted in the absence of any commercial or financial relationships that could be construed as a potential conflict of interest.

## Publisher’s Note

All claims expressed in this article are solely those of the authors and do not necessarily represent those of their affiliated organizations, or those of the publisher, the editors and the reviewers. Any product that may be evaluated in this article, or claim that may be made by its manufacturer, is not guaranteed or endorsed by the publisher.
